# Herpes zoster in patients with inflammatory arthritides or ulcerative colitis treated with tofacitinib, baricitinib or upadacitinib: a systematic review of clinical trials and real-world studies

**DOI:** 10.1007/s00296-022-05270-6

**Published:** 2023-01-13

**Authors:** Chrysoula G. Gialouri, Savvina Moustafa, Konstantinos Thomas, Emilia Hadziyannis, Dimitrios Vassilopoulos

**Affiliations:** 1 Joint Rheumatology Program, Clinical Immunology-Rheumatology Unit, 2nd Department of Medicine and Laboratory, National and Kapodistrian University of Athens, School of Medicine, General Hospital of Athens “Hippokration”, Athens, Greece; 2 4th Department of Internal Medicine, National and Kapodistrian University of Athens School of Medicine, Attikon University General Hospital, Chaidari, Greece

**Keywords:** JAK inhibitors, Herpes zoster, Rheumatoid arthritis, Spondyloarthritis, Ulcerative colitis, Systematic review

## Abstract

JAK inhibitors (JAKi) are new targeted-synthetic drugs, approved for various immune-mediated inflammatory diseases (IMIDs), including inflammatory arthritides (rheumatoid arthritis—RA, psoriatic arthritis—PsA, ankylosing spondylitis—AS) and ulcerative colitis (UC). JAKi have been associated with increased risk for herpes zoster (HZ), but the relative risk among different JAKi in these IMIDs remains unclear. We aimed to systematically review the incidence of HZ among RA, PsA, AS and UC patients treated with the approved doses of tofacitinib (TOFA), baricitinib (BARI) or upadacitinib (UPA). PubMed, Embase, Scopus, Cochrane and Web-of-Science were searched up to 30 March 2022. Clinical trials and real-world studies (RWS) were included. Outcomes assessed were the incidence rate (/100 patient-years) or/and cumulative incidence of HZ. From 1710 records, 53 clinical trials and 25 RWS were included (RA: 54, PsA: 8, AS: 4, and UC: 12). In clinical trials, the HZ-incidence was higher in TOFA-treated patients with RA (2.2–7.1/100 patient-years) or UC (1.3–7.6/100 patient-years) compared to PsA (1.7/100 patient-years), and with higher doses of TOFA in UC (10 mg/twice daily: 3.2–7.6/100 patient-years vs. 5 mg/twice daily: 1.3–2.3/100 patient-years). Evidence for HZ-risk in JAKi-treated patients with AS and in UPA-treated patients was limited. The HZ-incidence between TOFA and BARI groups in 2 RA RWS did not differ significantly. Concomitant glucocorticoid, but not methotrexate, use in RA increased the HZ-risk. This systematic review showed higher HZ-risk in RA or UC than PsA patients treated with TOFA, in those treated with higher TOFA doses or with concomitant glucocorticoids. Preventive measures and monitoring of JAKi-treated patients with IMIDs are essential in daily practice.

**Supplementary Information:**

The online version contains supplementary material available at 10.1007/s00296-022-05270-6.

## Introduction

Herpes zoster (HZ), caused by varicella zoster virus (VZV) reactivation, is a common cause of acute and occasionally long-term morbidity associated with increasing age, ethnicity, female sex, certain diseases such as diabetes mellitus and immunosuppressive therapies [1, 2]. In the general population, the incidence rate (IR) of HZ is 3–5/1000 person-years (PY), rising to more than 11/1000 PY in individuals > 80 years [1, 2].

Patients with immune-mediated inflammatory diseases (IMIDs) are more susceptible to develop HZ compared to the general population [3, 4]; this increased risk may in part be related to certain immune-suppressive/modulatory therapies, such as glucocorticoids (GCs) and cyclophosphamide [5–8]. Janus kinase inhibitors (JAKi), a new class of targeted-synthetic agents approved for the treatment of various IMIDs, have been also associated with increased HZ-risk [9].

JAKs are part of a 4-member cytoplasmic tyrosine kinase family (JAK1, JAK2, JAK3, and TYK2) which engage the intracellular domains of distinct cytokine and growth factor receptors and act by phosphorylating either themselves or adjacent molecules like activators of transcription (STATs) [10]. Thus, JAKi block the downstream signaling of a variety of pro-inflammatory cytokines and so modulate pivotal pathogenetic pathways operating in several IMIDs, including rheumatoid arthritis (RA), psoriatic arthritis (PsA), ankylosing spondylitis (AS) and ulcerative colitis (UC).

Tofacitinib (TOFA) is a JAK1/3 inhibitor, baricitinib (BARI) a JAK1/2 inhibitor, while upadacitinib (UPA) is predominantly a JAK1 inhibitor [10]. TOFA and UPA have been licensed for RA, PsA, AS and UC, while BARI only for RA (Supplementary Table-1). JAK/STATs apart from their role in downstream pro-inflammatory cytokine and growth factor signaling, participate also in interferon (IFN) signaling and thus in antiviral defense [11]. Signaling via JAK1 and JAK3 is also crucial for the differentiation, maturation and survival of CD4+ Th cells, CD8+ T cells and B cells, responsible for VZV control [12–14]. Moreover, the JAK/STAT pathway is involved in the development and activation of natural killer cells, whose deficiency predisposes to VZV infection [15].

At the same time, VZV life cycle is dependent on several JAK-family functions. VZV blocks the IFN-induced JAK/STAT signaling through inhibition of STAT1 and STAT2 and downregulation of JAK2 and IFN regulatory factor-9. In addition, VZV induces the STAT3 promoting the viral replication and spread into host tissues [16–18], as well modulates the antigen presentation on VZV-infected cells protecting them from CD4+ T-cell immune surveillance [19]. Therefore, JAKi may mimic the inhibitory action of VZV on IFN-induced JAK/STAT pathway, increasing the risk for VZV reactivation.

Studies underpin higher HZ-rates in IMID patients treated with JAKi than those receiving placebo or immunomodulating drugs [20–22]. Although HZ has been associated with all JAKi (“class effect”), it is unclear whether the risk differs across IMIDs. Furthermore, the role of concomitant therapies such as GCs or methotrexate (MTX) on HZ-risk has not been clarified.

We conducted a systematic literature review (SLR) of clinical trials and real-world studies (RWS) to: (i) evaluate the HZ-incidence among patients with RA, PsA, AS and UC after the initiation of treatment with the approved doses of 3 different JAKi (TOFA, BARI or UPA) and (ii) synthesize the available data for patient and treatment characteristics that could support the clinical management of HZ-risk.

## Methods

This SLR was undertaken according to the *Cochrane Handbook* [23] and *PRISMA 2020 statement* [24]. The review protocol was submitted in the International Prospective Register of Systematic Reviews (https://www.crd.york.ac.uk/prospero/display_record.php?ID=CRD42022323423) [25].

### Literature search strategy

PubMed, Embase, Cochrane-Library, Scopus and Web-of-Science were searched from inception up to 30 March 2022. JAKi of interest were tofacitinib-TOFA, baricitinib-BARI and upadacitinib-UPA. Our search strategy was guided from the PICO format: Population: RA, PsA, AS or UC patients; Intervention: treatment with TOFA, BARI or UPA; Comparator: placebo or active comparator [GCs, immuno-suppressants/-modulators] or none; Outcome: incident HZ events.

The search query was formed, in collaboration with an experienced librarian (ED), as follows: (“rheumatoid arthritis” OR “psoriatic arthritis” OR “ankylosing spondylitis” OR “spondyloarthritis” OR “ulcerative colitis”) AND (“Janus kinases inhibitors” OR “JAK inhibitors” OR “tofacitinib” OR “upadacitinib” OR “baricitinib” OR “DMARDs”) AND (“herpes zoster” OR “varicella zoster virus” OR “varicella zoster virus reactivation” OR “shingles”).

### Inclusion and exclusion criteria

Clinical trials (randomized and non-randomized) and RWS were included. We excluded: (a) case reports and case series with < 20 participants, editorials, letters, reviews, comments, surveys, recommendations, guidelines, expert opinions, and study protocols; (b) non-English studies; and (c) articles not referring to population/intervention/outcome of interest. SLRs, meta-analyses and pooled analyses were excluded from the final synthesis, but their references were screened to identify additional eligible publications. Articles and supplementary material non-available online were requested from the study investigators and were finally excluded if they were not provided up until the study submission.

### Study selection

All eligible studies had to fulfill the pre-specified PICO format. Two reviewers (CGG and SM) independently screened all records, in two steps: first, the titles and the abstracts were screened to determine relevance and excluded conference abstracts and then the full-texts to detect the adherence to inclusion criteria.

### Data extraction

Data extraction was performed independently by CGG and SM. First, CGG extracted data from all the eligible studies and hand-searched their reference lists to detect further suitable studies. Subsequently, SM independently and randomly extracted data from 25% of these studies (*n* = 20) and verified the rest 75%. Across all stages, any discrepancies were resolved by consensus or by a third reviewer (DV).

Review Manager 5.4 software was used to record the following data: (a) *study characteristics*: first author’s last name, publication year, study acronym, study type, country, number of participants in treatment arm(s), and observational period; (b) *patients’ characteristics*: disease, age, JAKi dose, comparators (when available), concomitant MTX and/or GC therapy, and baseline history of HZ; (c) *reports on incident HZ events*: cumulative incidence (%), incidence/event rate (IR/ER) per 100 patient-years (PY) with 95% confidence interval (CI) and/or hazard ratio (HR) (95% CI); and (d) *recurrence* of HZ during JAKi therapy.

### Data synthesis

Only data pertaining to groups treated with the approved JAKi doses per disease were analyzed (Supplementary Table-1). For the final synthesis, studies were clustered as per disease, class of JAKi and study type. The range of HZ-incidence in terms of cumulative incidence or IR/ER was estimated separately in clinical trials and RWS, including all the respective studies, regardless of the RoB.

### Risk of bias assessment

Risk of bias (RoB) was assessed independently by CGG and SM using the Cochrane RoB-2 tool [26] and the Newcastle–Ottawa-Scale [27, 28] for randomized and non-randomized studies, respectively. Discordances were openly discussed and if necessary DV made the final decision.

## Results

**Fig. 1:**
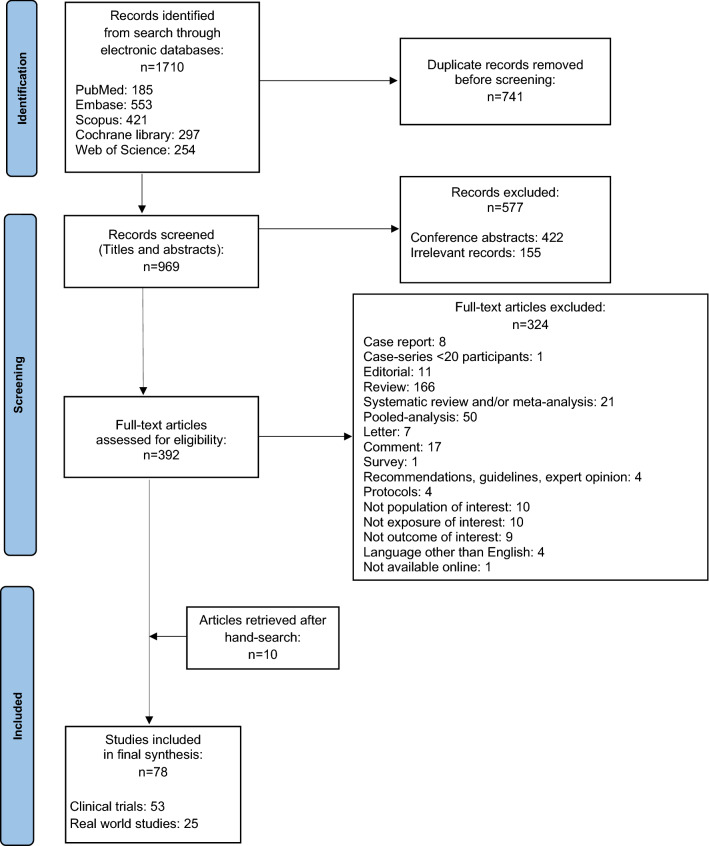
Flow-chart of the screening process performed for identification and selection of studies included in the systematic literature review

Our query initially retrieved 1710 references (Fig. 1). From them, after deduplication and titles/abstract screening, 392 were relevant to our search question and proceeded to full-text review. Finally, 68 articles met the inclusion criteria, in addition to 10 references identified after hand-search.

Agreement between reviewers in articles’ screening, validation of extracted data and RoB assessment was 91%, 97% and 85%, respectively.

### Characteristics of the included studies

Overall, 44 original articles of randomized controlled trials (RCT) were included (RA: 30, PsA: 6, AS: 4, and UC: 4), 9 of long-term extension studies (LTEs) (RA: 5, PsA: 1, and UC: 1) and 25 of RWS (RA: 19 and UC: 6). No RWS was identified for AS or UC patients.

Characteristics of the included studies are summarized in Supplementary Table-2 (RCTs and LTEs) and Supplementary Table-3 (RWS).

Most of the randomized studies included in this SLR (30/46) [44 RCTs and 2 LTEs with randomized arms at baseline [29–31]] had unclear RoB, attributed mainly to insufficient reporting on random sequence generation and allocation concealment. 8 RCTs showed high RoB; 6 included in their analysis an open-label period for participants/investigators and/or outcomes’ assessor [29, 32–36], 2 had selection bias in terms of the randomization process [33, 35] and 3 reported incompletely the outcome data [35, 37, 38] (see details in the Supplementary figure-1).

Among the 32 non-randomized studies (25 RWS and 7 LTEs), 5 had low, 20 intermediate and 7 high RoB (Supplementary table 4). RoB derived foremost from the absence of adjustments for confounders. In addition, in RWS high rates of study discontinuation (with ≥ 15% lost to follow-up deemed more likely to introduce bias) and/or inadequate follow-up assessment (defined for our outcome of interest as less than 1 year) were the most frequent bias-related issues.

## HZ-incidence after JAKi exposure

### Rheumatoid arthritis

#### Tofacitinib

In total, 23 RA studies for TOFA at the approved dose of 5 mg twice daily (BID) were eligible: 9 RCTs (10 original articles: 2 low, 7 unclear and 1 high RoB), 2 LTEs (intermediate RoB) (Supplementary Table-2) and 12 RWS (4 low, 5 intermediate, 3 high RoB) (Supplementary Table-3).

TOFA monotherapy was studied in 2 clinical trials [39, 40] and 1 RWS [41]. TOFA + MTX was administered in 1 RCT [42, 43] and 1 RWS [40, 41], while MTX was permitted in 2 clinical trials [44, 45] and 9 RWS [45–52]. Concomitant GCs were used in 8 RCTs [40, 53, 54] and 9 RWS [45–52].

The HZ-incidence in RCTs (within 12–96 weeks) ranged between 0 and 7.5% [38, 39, 42–44, 53–56]. In 2 longer duration studies (1 RCT and 1 LTE with 192- and 456-week duration, respectively), the HZ-incidence was higher at 12.4% and 10.7% [36, 40]. Of note, the IR was higher in a Japanese open-label LTEs (7.1/100 PY) compared to a global one (2.3/100 PY) [40, 45]. In RWS (mean observation period: 24–81.6 weeks), the incidence ranged between 1.3 and 16.7% and the IR between 1.4 and 6.5/100 PY [41, 46–52, 57–59]. Finally, in a post-marketing surveillance study for TOFA in RA, 7 HZ events out of 9291 adverse events were reported [60].

#### Baricitinib

9 RCTs (1 low, 3 unclear and 5 high RoB), 1 LTE (intermediate RoB) (Supplementary Table-2) and 9 RWS (1 low, 7 intermediate, 1 high RoB) (Supplementary Table-3) were retrieved for BARI 2 mg and/or 4 mg once daily (QD).

BARI combined with MTX was administered in 5 clinical trials [33, 34, 37, 61–64], MTX was permitted in 2 RCTs [65, 66] and 5 RWS [58, 59, 67–69], while GCs were used in 8 clinical trials [61–63, 65, 66] and 5 RWS [58, 59, 67–69].

The HZ-incidence in RCTs ranged between 0 and 1.7% for the 2 mg dose and between 1.3 and 7.0% for the 4 mg dose [33–35, 37, 61, 62, 64–66]. In 1 LTE (4 mg QD), the incidence was 5.6% the first 76 weeks and decreased to 1.3% between 76 and 128 weeks [63]. In RWS (follow-up: 24–48 weeks), the HZ-incidence in groups receiving 4 mg QD ranged between 1.3 and 4.9% [58, 67, 68]. In another 2 cohorts, the pooled analysis of both BARI dose-groups revealed an incidence of 3.6% and 6.2%, respectively [59, 69]. Finally, a post-marketing surveillance study in RA found 49 HZ cases out of 1598 reported adverse events [70].

#### Upadacitinib

For UPA, 12 primary reports of RCTs (1 low, 9 unclear and 2 high RoB) and 1 LTE (unclear RoB) were included (Supplementary Table-2). UPA combined with MTX was studied in 1 RCT [71], while MTX was permitted in 5 clinical trials [30, 72–76].

The range of HZ-incidence in RCTs (12–48 weeks) was 0.5–2.2% [29, 32, 71–78], whereas in a subgroup analysis of Japanese patients from a 24-week RCT, the incidence was higher at 7.4% [79]. The overall HZ-incidence and ER in a long-term trial (within 108 weeks) were 6.1% and 3.1/100 PY, respectively [29], while in another Japanese LTEs (84-week duration), the incidence and IR were likewise higher (21.9% and 12.3/100 PY, respectively) [30, 74].

## Psoriatic arthritis

### Tofacitinib

3 RCTs (low RoB) and 1 LTE (intermediate RoB), but no RWS, were eligible for TOFA in PsA (Supplementary Table-2). In 3 of them, MTX and/or GCs were co-administered [80–82]. In RCTs (12–48-week duration), the HZ-incidence ranged between 0 and 3.3% [80, 81, 83] with a similar incidence (2.5%) in a LTE (144 weeks) [82].

### Upadacitinib

Evidence for HZ under UPA therapy were provided by 2 RCTs (unclear RoB). The HZ-incidence ranged between 0.9 and 1.4% (up to week 24) [84, 85] while the respective ER in 1 study was 3.8/100 PY (up to week 56) [86]. RWS relevant to our PICO format were not found.

## Ankylosing spondylitis

### Tofacitinib

Overall, 2 RCTs (unclear RoB) were included for TOFA in AS patients. In the first, no HZ event was documented up to 12 weeks on treatment followed by 4 weeks of follow-up [87], while in the second trial, an incidence of 0%, 1.5% and 2.3% was reported at weeks 16, 32 and 48 on treatment, respectively [88]. No RWS was identified.

### Upadacitinib

Data for HZ during UPA therapy in AS patients were retrieved only from 1 RCT (2 original articles: 1 low and 1 unclear RoB). The interim analysis (up to week 64) indicated an incidence of 2.5% [89]. RWS were not found.

## Ulcerative colitis

### Tofacitinib

Overall, 5 RCTs (2 low and 3 unclear RoB), 1 LTE (intermediate RoB) (Supplementary Table-2) and 6 RWS (3 low and 3 high RoB) (Supplementary Table-3) were eligible. GCs were permitted in 5 clinical trials [90–92] and 4 RWS [93–96]. TOFA monotherapy was studied in 1 RCT [97] and 1 RWS [98].

In RCTs (8–52 weeks), the HZ-incidence ranged between 0.5 and 5.1% for the 10 mg BID dose [59, 61, 62] and 1.4–1.5% for the 5 mg BID dose [91, 97]. In the LTEs (up to week 240), the respective incidence for these doses was 7.8% and 7.4% [92]. Similarly, in 1 RCT and 1 LTE, the IRs for the 10 mg BID dose were higher, at 3.2 and 3.6/100 PY, compared to 1.3 and 2.1/100 PY for the 5 mg BID dose [92, 97]. In addition, Vermeire et al. randomized patients at remission, who had received TOFA 10 mg BID for ≥ 2 consecutive years, either to remain at the 10 mg BID dose or to de-escalate to 5 mg BID. Analyses up to week 24 showed higher HZ-rates in the 10 mg BID group compared to the 5 mg BID group (4.3% vs. 1.4%) with IRs of 3.2/100 PY and 1.3/100 PY, respectively [97].

In RWS (mean follow-up: 24–60 weeks), the pooled HZ-incidence ranged from 0 to 7.9% [93–96, 98, 99]. Interestingly, across all of them, 9 of the 13 (69%) HZ cases occurred in patients receiving the high TOFA dose (10 mg BID).

### Upadacitinib

Limited evidence is available for UPA in AS, since only 1 RCT (unclear RoB), and none RWS, was eligible. Data from the first 8 weeks on treatment showed an HZ-incidence of 0% for the 15 mg and 30 mg groups and 0.8% for the 45 mg group [100].

## Comparative studies of HZ-incidence among different JAKi

We identified only 2 RWS in Japanese RA patients at which the HZ-rate was compared between TOFA and BARI groups (Table 1). Iwamoto et al. demonstrated a slightly higher HZ-incidence in TOFA than BARI group (5.6% vs. 4.9%) [58]. Inversely, Miyazaki et al. found higher incidence for BARI (3.6% vs. 1.3%) [59]. However, in both studies, the differences were non-significant. Head-to-head studies comparing the HZ-incidence among different JAKi were not found.

**Table 1:** Studies included in this SLR, which reported the difference on the HZ-incidence among patients receiving different JAKi

Author (ref), year, region	Study type	Observational period	Age, years^a^	Concomitant GCs ^b^ % (dose^c^)	Cumulative HZ-incidence, % (n/N)		*p*-value	RoB
					TOFA	BARI		
Miyazaki [59] 2021, Japan	Cohort	24 weeks	59.1 (13.4)	TOFA^*^: 16% (5.0) BARI^*^: 17% (7.5)	1.3^¥^ (2/156)	3.6^¥¥^ (5/138)	0.18	Intermediate
Iwamoto [58] 2021, Japan	Cohort	24 weeks	66.5 (12.2)	TOFA^*^: 53% (4.8) BARI^*^: 47% (4.8)	5.6^§^ (9/161)	4.9^§§^ (4/81)	NR^¶^	Intermediate

^a^Mean/median (SD/IQR)

^b^Reported at baseline

^c^Mean daily dose (mg)

^*^Non-significant difference of concomitant GC use between TOFA and BARI groups

^¥^ 89.7% received 10 mg and 10.3% received 5 mg BID

^¥¥^ 88.4% received 4 mg and 11.6% 2 mg QD

^§^All received 5 mg BID

^§^^§^All received 4 mg QD

^¶^ Not reported. The authors only mentioned that the difference was non-significant

*GCs* glucocorticoids, *HZ* herpes zoster, *RoB* risk of bias, *TOFA* tofacitinib, *BARI* baricitinib

## The effect of concomitant MTX and/or GCs on HZ-incidence

In 1 LTE with 1123 RA patients, the IR of HZ was similar between TOFA monotherapy and TOFA combined with MTX (2.4 vs. 2.2/100 PY) [40]. In line, Curtis et al*.* analyzed 8030 RA patients from a US insurance database and found only a small difference in HZ-rates between those treated with TOFA vs. TOFA and MTX (3.7 vs. 3.4/100 PY) [41]. Similar results were reported in an Italian study [67]. On the other hand, 1 RCT with PsA patients reported a higher incidence (up to week 48) in the combination than the monotherapy group (2.2% vs. 1.1%) [83].

In contrast, data from RA studies have shown that concomitant GC use increases the HZ-risk (Table 2). Curtis et al*.* in the same study showed that the addition of GCs increased the HZ-risk in both TOFA monotherapy and combination groups (TOFA: 3.7/100 PY and TOFA + MTX: 3.4/100 PY vs. TOFA + GCs: 6.0/100 PY and TOFA + MTX + GCs: 6.5/100 PY) [41]. In addition, in a cohort of 446 RA patients, HZ was the most frequent infection reported among BARI-treated patients under concomitant GCs compared to other infections [67].

**Table 2:** Studies included in this SLR that examined the role of concomitant glucocorticoids on the risk of HZ development in JAKi-treated patients

Author, year (ref), country	Study type follow-up	Disease JAKi	Age, years^a^	Key findings			RoB
Curtis 2019 [41] US	Cohort NR	*RA* TOFA	60.3 (12.6)		IR (95% CI)*	HR (95% CI)	Low
				TOFA monotherapy	3.7 (2.9–4.6)	1.0 (ref)	
				TOFA + MTX	3.4 (2.3–5.0)	0.99 (0.64–1.54)	
				TOFA + GCs	6.0 (4.9–7.5)	1.75 (1.28–2.41)	
				TOFA + MTX + GCs	6.5 (4.8–8.8)	1.96 (1.33–2.88)	
Guidelli 2021 [67] Italy	Prospective cohort ≤ 48 weeks	*RA* BARI	59.0 (11.9)	In BARI-treated patients on GCs, the rate of VZV reactivation was significantly higher compared to other infections (83% vs. 25%; p = 0.034)			Intermediate
Redeker 2022 [101] Germany	Prospective cohort ≤ 480 weeks	*RA* TOFA, BARI or UPA	57.9 (12.5)	Among JAKi-treated patients, the ER of HZ was comparable between those with and those without concomitant GC use Dose-dependent relationship; HR (95% CI): GCs 5–10 vs. 0 mg/day: 1.24 (0.98 to 1.57) GCs > 10 vs. 0 mg/day: 3.45 (2.14 to 5.55)			Intermediate

^a^Mean/median (SD/IQR)

*Per 100 patient-years

*ref* reference, *NR* not reported, *ER* event rate, *IR* incidence rate, *HR* hazard ratio, *CI* confidence interval, *JAKi* Janus kinases inhibitor, *TOFA* tofacitinib, *BARI* baricitinib, *UPA* upadacitinib, *RoB* risk of bias, *VZV* varicella zoster virus, *GCs* glucocorticoids

This enhanced HZ-risk in GC-treated patients could be partially dose-dependent. Indeed, in a large prospective cohort of JAKi-treated patients with RA, although the ER of HZ was comparable between patients with and those without concomitant GC use, the adjusted HZ-risk was significantly increased in patients on a “high” GC dose (> 10 mg/day), but not in those on a “low” GC dose (5–10 mg/day), compared to patients who did not receive GCs [101].

## Different risk of HZ between JAKi and non-biologic/biologic immunomodulators

In 5 RA trials, the HZ-incidence in patients treated with TOFA, BARI or UPA was higher compared to MTX [39, 42, 61, 78]. Similarly, 1 RA RWS showed a 3.6-fold increased risk of HZ with JAKi compared to conventional-synthetic disease-modifying anti-rheumatic drugs (csDMARDs) [101].

Regarding biologics, a higher HZ-rate was found for JAKi compared to TNFi in 3 RA and 2 PsA head-to-head RCTs [29, 36, 37, 81, 85, 101]. In addition, 5 RA RWS studies showed a higher risk for TOFA compared to various biologics [47, 48, 50, 51, 101]. However, in a Korean study with RA patients treated with JAKi or biologics, after adjustment for disease duration, number of previous targeted therapies, concomitant MTX and/or GC use, the difference was attenuated (7.6% vs. 4.8%) [102]. Conversely, in 3 RA RCTs, the incidence was similar between TOFA/UPA and ADA/ABA groups [53, 54, 75]. More details are given in Supplementary Tables 2 and 3.

## Association of previous history of HZ with a new HZ episode after JAKi exposure

Among the included studies, only 2 clinical trials and 4 RWS provided information about patients’ previous HZ history. In 5 of them, less than 15% of patients who developed HZ during JAKi treatment had history of an HZ event [30, 34, 47, 57, 67, 94, 103]. In contrast, in a cohort with 125 RA patients, 100% (7/7) of patients who developed HZ during TOFA treatment had HZ history compared to 14.4% (17/118) of those who did not develop HZ during TOFA therapy (p < 0.001)[57].

## Recurrence of HZ during JAKi therapy

Limited data are available for the rate of HZ recurrence in patients who develop the first HZ episode during JAKi therapy. In the ORAL Sequel trial, an open-label LTE with 4481 RA patients, HZ recurrence was 2.6/100 PY within 456 weeks [40]. Moreover, in a small cohort with 29 RA patients who had previously experienced an HZ event during TOFA or BARI therapy, only 1 patient developed a second episode within a median follow-up of 12 months. Interestingly, in this dataset, the continuation or resumption (after completion of HZ therapy) of the same JAKi did not seem to affect the risk of HZ recurrence [103].

## Discussion

In this SLR, we reviewed the HZ-incidence among patients with different IMIDs (RA, PsA, AS and UC) who were treated with different JAKi (TOFA, BARI or UPA). These results provide a better understanding of the relative HZ-risk across the expanding spectrum of JAKi indications and inform physicians and patients alike for the necessary steps for its prevention.

In RA, the overall HZ-rates in RCTs ranged between 0 and 12.4% for TOFA [36, 39, 40, 42–44, 53–56], 0–7% for BARI [33–35, 37, 61–66] and 0.5–7.4% for UPA [29, 30, 32, 71–79, 104]. Similar rates were reported in RWS: TOFA: 1.3–16.7% [41, 46–50, 52, 57] and BARI: 1.3–6.2% [58, 59, 67–69]] (Table 3).

**Table 3:** Incidence of herpes zoster reported in the included RCTs, LTEs and RWS

Disease	JAKi	Dose	RCTs		LTEs		RWS	
			Cumulative incidence % [weeks^¥^]	IR/ER^*^ (/100 PY)	Cumulative incidence % [weeks^¥^]	IR/ER (/100 PY)	Cumulative incidence % [weeks^¥^]	IR/ER (/100 PY)
RA	TOFA	5 mg BID	0–7.5% [12–96] 12.4% [192^§^]	**–**	10.7% [456]	2.2–7.1	1.3–16.7% [24–81.6^§^]	1.3–6.5
	UPA		0.5–7.4% [12–48]	2.3^*^–3.1^*^	6.1–21.9% [84–108]	3.1–12.3	–	–
	BARI	2 mg QD	0–1.7% [12–24]	–	–	–	1.3–6.2% [24–48] (*2 or 4 mg QD*)	8.4 (*2 or 4 mg QD*)
		4 mg QD	1.3–2.3% [12–28] 7% [50]	–	1.3–5.6% [52]	1.3–5.8		
PsA	TOFA	5 mg BID	0–3.3% [12–48]	**–**	2.5% [144]	1.7	–	–
	UPA	15 mg QD	0.9–1.4% [24]	3.8^*^	–	–	–	–
AS	TOFA	5 mg BID	0–2.3% [16–48]	–	–	–	–	–
	UPA	15 mg QD	2.5% [64]	2.1^*^	–	–	–	–
UC	TOFA	5 mg BID	1.4–1.5% [24–52]	1.3	4.5–7.4% [48–180]	2.1–2.3	0–7.9% [24–60^§^] (*5 or 10 mg BID*)	–
		10 mg BID	0.5–5.1% [8–52]	3.2	7.8–12.3% [48–180]	3.6–7.6		–
	UPA	15 mg QD	0% [8]	–	–	–	–	–
		30 mg QD	0% [8]	–	–	–	–	–
		45 mg QD	0.8% [8]	–	–	–	–	–

^**¥**^Observational period

^§^Mean/median

*RCT* randomized controlled trial, *LTEs* long-term extension studies, *RWS* real-world study, *RA* rheumatoid arthritis, *PsA* psoriatic arthritis, *AS* ankylosing spondylitis, *UC* ulcerative colitis, *TOFA* tofacitinib, *BARI* baricitinib, *UPA* upadacitinib, *BID* twice daily, *QD* once daily, *IR* incidence rate, *ER* event rate, *PY* patient-years

*ER/100 PY

For PsA and AS, in general, the rates were lower for the 2 approved JAKi (TOFA, UPA); however, data were limited and mainly derived from RCTs. More specifically, in PsA patients, the HZ-rate for TOFA was 0–3.3% [80–83] and for UPA 0.9–1.4% [84, 85]. The respective rates in AS patients were 0–2.3% for TOFA [87, 88] and 2.5% for UPA [89, 105] (Table 1). Long-term trials and RWS are required to investigate the real HZ-incidence under JAKi therapy in these patient populations.

Finally for UC, more studies are available for TOFA, showing a higher HZ-rate for the higher dose [10 mg BID; RCTs: 0.5–5.1% [90, 91, 97], LTEs: 7.8–12.3% [92, 106]] compared to the lower [5 mg BID; RCTs: 1.4–1.5% [91, 97], LTEs: 4.5–7.4% [92, 106]].

From indirect comparisons of TOFA clinical trials, we appraised that HZ-incidence was similar between RA (2.2–7.1/100 PY) and UC patients (1.3–7.6/100 PY), but lower in PsA (1.7/100 PY) patients. Higher age of RA patients, more frequent GC use and/or higher TOFA doses in UC patients, could explain this finding. In addition, differences in underlying disease mechanisms may play a role. In accordance, Burmester et al*.* in a pooled analysis of RA, PsA and UC RCTs and LTEs found higher IRs in RA and UC than PsA cohorts. This difference was attributed to the highest proportion of Asian patients enrolled in RA and UC studies and/or to concomitant GC use [107].

In the absence of head-to-head studies comparing the HZ-risk among different JAKi, we looked for evidence from non-randomized studies for an indirect comparison. In 2 Japanese RA observational studies, there were non-significant differences in the HZ-incidence between TOFA and BARI groups. Similar results were reported in recent meta-analyses of RA clinical trials that compared the BARI- and UPA-related HZ-risk [20, 107, 108]. Conversely, another meta-analysis of clinical trials and RWS on patients with different IMIDs showed a higher pooled IR of HZ for UPA (3.92/100 PY), followed by BARI (2.16/100 PY) and TOFA (1.62/100 PY) therapy [109]. Notably, in this study, results were not stratified per type of IMID and so comparisons were not feasible.

Another important question is whether there is a dose-dependent increase of HZ-risk in JAKi-treated patients. In the included clinical trials, we found a higher IR of HZ with the high dose of TOFA in UC (10 mg BID: 3.2–7.6/100 PY vs. 5 mg BID: 1.3–2.3/100 PY). To compare the BARI doses in RA, only the cumulative HZ-incidence was available from the short-term RCTs (up to 28-week duration); the HZ-rate was slightly higher with the high dose (4 mg QD: 1.3–2.3% vs. 2 mg QD: 0–1.7%) (Table 1). Accordingly, in a meta-analysis of 4 BARI RCTs, which have been included in this SLR, the HZ-rate was estimated to be similar between the two doses up to week 12, but higher with 4 mg vs. 2 mg QD at 24 weeks’ follow-up. However, this difference was non-significant [110]. In addition, there are conflicting data for TOFA dosing in RA, with 1 meta-analysis showing similar HZ-rates between TOFA 5 mg and 10 mg BID [111], whereas in a pooled analysis of RA RCTs by Winthrop et al., the TOFA dose was an independent risk factor for HZ [112].

Ethnicity appears to be also an important risk factor for HZ. In general, we found higher HZ-rates in Japanese patient-groups treated with JAKi. So far, there is not a clear explanation for this finding. A recent meta-analysis of genome-wide association studies revealed multiple loci associated with faster onset of HZ in European and East Asian patients with RA or PsO treated with TOFA [113].

As for the role of concomitant therapies, we identified GCs as an additional risk factor for HZ in several RA studies, with some of them displaying a dose-dependent relationship. In contrast, MTX did not appear to confer additional risk in RA. In agreement, Winthrop et al*.* in a pooled analysis of 6192 RA demonstrated that concomitant GCs, at any dose, independently increased the HZ-risk, whereas the addition of csDMARDs (mainly MTX) had no effect when compared to TOFA monotherapy [112]. Similar results for the concomitant GC use have been reported for UC patients within the TOFA clinical program [114], as well for PsA patients in a previous SLR [115].

In general, JAKi when used as monotherapy displayed higher HZ-rates compared to non-biologics (mainly MTX) in RA and biologics in RA and PsA. In accordance, the IR of HZ in a previous SLR was estimated between 3–4/100 PY (in Western Europe, USA, Australia) and 9/100 PY (in Japan, Korea) with JAKi vs. 2–3/100 PY with TNFi [116]. However, more well-designed studies are needed to examine whether these differences are influenced by confounders.

The timing of HZ development after JAKi initiation could be clinically relevant. We observed that in RA trials for TOFA, the HZ-incidence was higher in longer duration studies (> 96 weeks); however, the timepoint of HZ occurrence was not reported. In addition, IRs were higher in LTEs than RCTs for TOFA in UC patients. Studies for PsA were limited, but the HZ-incidence in one LTE was within the range reported in RCTs. In addition, in SELECT-COMPARE trial, the IR did not differ between 48- and 108-week durations of UPA therapy [29, 71]. In line, pooled analyses of RA and UC clinical trials have indicated that longer duration of TOFA exposure did not increase the HZ-risk [114, 117]. More details were provided by RWS. In 3 cohorts, most events occurred within the first 16 weeks of JAKi initiation [57, 67, 98], while in another 4 cohorts, the median/mean time to VZV reactivation since JAKi commencement ranged between 7 and 44 weeks [49, 69, 94, 103].

Thus far, studies examining whether patients with HZ history have a higher risk for HZ recurrence after JAKi initiation are lacking. Of note, most clinical trials excluded patients with a history of > 1 HZ episode or a single episode of disseminated HZ. Thus, the association of HZ history with a new HZ episode after exposure to JAKi was examined only in 1 RA cohort at which was found to be significant [57]. In accordance, an integrated analysis of the SELECT program for UPA in RA showed that the history of HZ was a significant risk factor for incident HZ (HR: 24.19; 95% CI, 15.94–36.72) [118].

The risk of HZ recurrence after the first episode during JAKi treatment is also unclear. Data from the included studies are scarce, but it seems that the HZ recurrence is rare even in IMID patients. In a large RA trial for TOFA, the recurrence rate was 2.6/100 PY [40]. In addition, Cohen et al. in a post hoc analysis of clinical trials found that about 5% (36/783) of PsA and 11% (783/7061) of RA patients had at least 1 HZ event, but only 3% (1/36) and 8% (63/783), respectively, had more than 2 events during TOFA therapy [119].

The availability of vaccines against HZ is a crucial development for the prevention of therapy-related HZ in patients with IMIDs. Data regarding the efficacy and safety of the older, live attenuated and of the most recently available, non-live recombinant zoster vaccine are scarce [120]. In this SLR, the rate of vaccination against HZ at baseline was reported in a few clinical trials [30, 40, 54, 73, 77, 78] and RWS [41, 47, 51, 93, 94, 102, 103], and it was generally low, less than 10%. Notably, in a retrospective analysis of US insurance databases for TOFA-treated RA patients, prior vaccination with a live-attenuated zoster vaccine was associated with a trend towards lower HZ-risk (HR: 0.60; 95% CI 0.34–1.05)[41]. Thus, meaningful conclusions about the protective role of zoster vaccination in JAKi-treated patients cannot be made.

In the 2019 EULAR recommendations, HZ vaccination was recommended for high-risk patients with IMIDs [120], while the 2022 Guidelines from the Advisory Committee on Immunization Practices of the United States Centers for Disease Control and Prevention recommend the use of the newer recombinant zoster vaccine for patients ≥ 19 years who are or will be immune-deficient/suppressed because of disease or therapy [121]. The new recombinant vaccine, which has been licensed in Europe since March 2018, has shown moderate to high efficacy and an acceptable safety profile in immunocompromised persons and is thus appropriate for patients on high-risk for HZ such as those treated with JAKi for IMIDs.

Most of the HZ events that occurred during JAKi therapy were classified as non-serious and involved 1 or 2 adjacent dermatomes both in clinical trials and RWS. Furthermore, we observed discrepancies in the management of JAKi treatment after HZ development, mainly in RWS, given that some RCTs required discontinuation of treatment in case of HZ occurrence [34, 122]. Even so, for most of the cases, JAKi therapy was continued after HZ resolution. Similarly, in a recent post hoc analysis of 21 RA and 3 PsA clinical studies, the majority of first or recurrent HZ events were non-serious, mild, or moderate in severity and clinically manageable with antiviral therapy and/or with temporary drug discontinuation [119]. Recently, the EULAR expert committee proposed temporarily interruption of JAKi therapy until the HZ episode is resolved, while antiviral prophylaxis could be considered in individuals with recurrent zoster [116].

This study has some limitations. First, an accurate estimate of the relative HZ-risk among different IMIDs and different JAKi cannot be provided, given that this requires a meta-analysis which was out of our scope. Second, in this SLR, many of the included studies had unclear/intermediate RoB. However, in RCTs, this derived mainly from insufficient reporting of randomization procedure that we consider not to significantly influence our outcomes. Third, to assess the RoB in non-randomized studies, we implemented the Newcastle–Ottawa-Scale, which although is not the most powerful tool, is suggested from *Cochrane handbook* for non-Cochrane Reviews [123]. Besides, we still found at least intermediate RoB in the majority of the included non-randomized studies, and so the quality of them was not overestimated. Fourth, the true impact of concomitant MTX and/or GC use on HZ-risk could not be estimated since neither doses nor duration of exposure were reported in detail. Finally, some studies (mainly RWS) provided pooled data from groups receiving different JAKi and/or doses, thus the HZ-incidence with specific JAKi/dose could not be ascertained.

This SLR has also important strengths. We searched 5 electronic databases covering the largest part of the literature. Importantly, aside from RCTs and LTEs, we also included RWS most of which have recently been published and so have not been reviewed previously. In addition, we analyzed data referring only to the approved JAKi doses in order to provide a useful overview applicable to daily clinical practice. In addition, the search strategy was supported by an experienced librarian, while the screening process and RoB assessment were fully and independently conducted by two reviewers. Finally, the quality of studies was assessed using reliable tools recommended for SLRs.

## Conclusion

This SLR provides useful updated information regarding the incidence and management of HZ during JAKi treatment in patients with IMIDs. Overall, this evidence supports that HZ-risk is a “class” effect of JAKi (higher risk compared to other non-biologic and biologic drugs) and is partially disease- (higher risk for TOFA-treated patients with RA or UC compared to PsA) and dose- (higher risk with the higher dose of TOFA) dependent, while concomitant GC use remains an additional risk factor. It is reassuring that most episodes were non-serious and JAKi therapy could be continued without consequences. These findings are instructive for the optimal use of JAKi and the management of HZ-risk in clinical practice, highlighting the need for more real-world data on the efficacy and safety of the newly available HZ vaccines, as well for the comparative HZ-risk among different JAKi.

## Supplementary Information

Below is the link to the electronic supplementary material.

**Supplementary file 1**: Table 1 Approved doses of tofacitinib, baricitinib and upadacitinib for the treatment of RA, PsA, AS and UC. Table 2 RCTs and LTEs included in this SLR. Study and patient characteristics, incident herpes zoster events and risk of bias. Table 3 Real-world studies included in this SLR. Study and patient characteristics, incident herpes zoster events and risk of bias. Table 4 Risk of bias in non-randomized studies according to the Newcastle-Ottawa scale. Figure 1 Risk of bias in randomized studies according to the Cochrane RoB 2 tool.

## Data Availability

Data for this study are presented in the manuscript, tables and supplementary material. Additional data are available upon request to the corresponding author.
