# Supplementary material for: Herpes zoster in patients with inflammatory arthritides or ulcerative colitis treated with tofacitinib, baricitinib or upadacitinib: a systematic review of clinical trials and real-world studies
Source: Rheumatol Int. 2023 Jan 13;43(3):421–35. doi: 10.1007/s00296-022-05270-6 (PMC9968274; doi:10.1007/s00296-022-05270-6)
Supplement: Supplementary file 1 — Table 1 Approved doses of tofacitinib, baricitinib and upadacitinib for the treatment of RA, PsA, AS and UC. Table 2 RCTs and LTEs included in this SLR. Study and patient characteristics, incident herpes zoster events and risk of bias. Table 3 Real-world studies included in this SLR. Study and patient characteristics, incident herpes zoster events and risk of bias. Table 4 Risk of bias in non-randomized studies according to the Newcastle-Ottawa scale. Figure 1 Risk of bias in randomized studies according to the Cochrane RoB 2 tool. [file 296_2022_5270_MOESM1_ESM.docx]

**Supplementary material**

**Supplementary Table-1** Approved doses of tofacitinib, baricitinib and upadacitinib for the treatment of RA, PsA, AS and UC.

|  | **Tofacitinib** | **Baricitinib** | **Upadacitinib** |
| --- | --- | --- | --- |
| **RA** | 5 mg BID | 2 mg or 4 mg QD | 15 mg QD |
| **PsA** | 5 mg BID | - | 15 mg QD |
| **AS** | 5 mg BID | - | 15 mg QD |
| **UC** | 10 mg BID  (induction therapy for ≥8 weeks);  then 5 mg or 10 mg BID  (maintenance therapy) | - | 45 mg QD  (induction therapy for ≥8 weeks);  then 15 mg or 30 mg QD^§^  (maintenance therapy) |

^§^ The recommended maintenance dosage is 15mg QD. 30mg QD may be considered for patients with refractory, severe, or extensive disease.

Baricitinib has been approved only for RA.

RA; rheumatoid arthritis, PsA; psoriatic arthritis, AS; ankylosing spondylitis, UC; ulcerative colitis, BID; twice daily, QD; once daily.

**Supplementary Table-2** RCTs and LTEs included in this SLR. Study and patient characteristics, incident herpes zoster events and risk of bias.

| **Author, year (ref)** | **Study type** | **Region** | **Age, years^a^** | **Observational period, weeks** | **Treatment groups** | **Concomitant**  **MTX and/or**  **GCs**^§^**, %** | **Herpes zoster** | | **RoB** |
| --- | --- | --- | --- | --- | --- | --- | --- | --- | --- |
|  |  |  |  |  |  |  | **Cumulative incidence, % (n/N)** | **Incidence or event rate^c^** |  |
| **Rheumatoid arthritis** | |  |  |  |  |  |  |  |  |
| **TOFACITINIB 5mg BID** | |  |  |  |  |  |  |  |  |
| Fleischmann 2012 (38)  (ORAL Solo) | RCT  Phase 3 | NA, LA, EU, Asia | 51.4  (11.9) | 0-24 | TOFA 5 mg or 10mg BID | MTX: 0, GCs: 59 | 0.2 (1/488) ^§§^ | NR | High |
|  |  |  |  |  | PBO switched at week 12 to TOFA | MTX: 0, GCs: 63 | 0.0 (0/61) |  |  |
| Fleischmann 2017 (54)  (ORAL Strategy) | RCT  Phase 3b/4 | Global | 50.1  (13.0) | 24-48 | TOFA | GCs: 58 | 1.0 (4/384) | NR | Unclear |
|  |  |  |  |  | TOFA + MTX | GCs: 57 | 2.1 (8/376) |  |  |
|  |  |  |  |  | ADA + MTX | GCs: 57 | 1.6 (6/386) |  |  |
| Kremer 2013 (44)  (ORAL Sync) | RCT  Phase 3 | Global | 51.8 (11.5) | 96 | TOFA | MTX: 79, GCs: 60 | 0.3 (1/315) | NR | Unclear |
|  |  |  |  |  | PBO switched at week 12 or 24  to TOFA |  | 0.0 (0/79) |  |  |
| Kremer 2009 (55) | RCT  Phase 2a | NA, LA, EU | 49.6 (11.5) | 12^†^ | TOFA | MTX: 0, GCs: 64 | 1.6 (1/61) | NR | Unclear |
|  |  |  |  |  | PBO | MTX: 0, GCs: 62 | 1.5 (1/65) |  |  |
| Lee 2014 (39)  (ORAL Start) | RCT  Phase 3 | Global | 50.0 | 96 | TOFA monotherapy |  | 3.5 (13/373) | NR | Low |
|  |  |  |  |  | MTX monotherapy |  | 1.1 (2/186) |  |  |
| Tanaka 2015 (56) | RCT  Phase 2 | Japan | 53.0 (11.2) | 12 | TOFA | MTX: 0, GCs: NR | 1.9 (1/52) | NR | Low |
|  |  |  |  |  | PBO |  | 0.0 (0/52) |  |  |
| van der Heijde 2013 (42)  (ORAL Scan) | RCT  Phase 3 | NA, LA, EU, Asia, Oceania | 53.5 (11.6) | 12 | TOFA + MTX | GCs: 0 | 0.9 (3/321) | NR | Unclear |
|  |  |  |  |  | PBO + MTX |  | 0.0 (0/160) |  |  |
|  |  |  |  | 12-24 | TOFA + MTX |  | 1.9 (6/321) |  |  |
|  |  |  |  |  | TOFA + MTX switched from PBO + MTX |  | 0.0 (0/42) |  |  |
|  |  |  |  |  | PBO + MTX |  | 0.0 (0/81) |  |  |
|  |  |  |  | 24-48 | TOFA + MTX |  | 1.9 (6/321) |  |  |
|  |  |  |  |  | TOFA + MTX switched from PBO + MTX |  | 4.9 (4/81) |  |  |
| van der Heijde 2019 (43) (ORAL Scan) |  |  |  | 48-96 | TOFA + MTX |  | 2.8 (9/321) |  | Unclear |
|  |  |  |  |  | TOFA + MTX switched from PBO + MTX |  | 0.0 (0/81) |  |  |
| van Vollenhoven 2012 (53)  (ORAL Standard) | RCT  Phase 3 | Global | 53.7 (12.4) | 0-12 | TOFA + MTX | GCs: 62 | 0.0 (0/204) | NR | Unclear |
|  |  |  |  |  | PBO + MTX | GCs: 66 | 0.0 (0/108) |  |  |
|  |  |  |  |  | ADA + MTX | GCs: 61 | 0.0 (0/204) |  |  |
|  |  |  |  | 12-24 | TOFA + MTX |  | 1.0 (2/204) |  |  |
|  |  |  |  |  | TOFA + MTX switched from PBO + MTX |  | 0.0 (0/28) |  |  |
|  |  |  |  |  | PBO + MTX |  | 1.7 (1/59) |  |  |
|  |  |  |  |  | ADA + MTX |  | 0.5 (1/204) |  |  |
|  |  |  |  | 24-48 | TOFA + MTX |  | 2.0 (4/204) |  |  |
|  |  |  |  |  | TOFA + MTX switched from PBO + MTX | GCs: 73 | 0.0 (0/52) |  |  |
|  |  |  |  |  | ADA + MTX |  | 2.0 (4/204) |  |  |
| Wollenhaupt 2019 (40)  (ORAL Sequel) | Open-label LTEs | Global | 53.3 (11.7) | 456 | TOFA |  | 10.7 (120/1123) | IR 2.3 (2.3-3.3) | Intermediate |
|  |  |  |  |  | TOFA monotherapy |  | 9.5 (29/305) | IR 2.4 (1.6-3.5) |  |
|  |  |  |  |  | TOFA + csDMARDs (mainly MTX) | MTX: NR, GCs: NR | 8.3 (52/630) | IR 2.2 (1.7-2.9) |  |
| Yamanaka 2016 (45) | Open-label LTEs | Japan | 53.5  (11.2) | 288 | TOFA | MTX: 51, GCs: 67 |  | IR 7.1 (5.5-8.9) | Intermediate |
| Ytterberg 2022 (36)  (ORAL Surveillance) | RCT  Phase 3b/4 | Global | 61.1  (7.2) | 192  (median) | TOFA + MTX | GCs: 58 | 12.4 (180/1455) | NR | High |
|  |  |  |  |  | TNFi (ADA or ETN) + MTX | GCs: 57 | 4.0 (58/1451) |  |  |
| **BARICITINIB 2mg or 4mg QD** | |  |  |  |  |  |  |  |  |
| Dougados 2016 (65)  (RA-BUILD) | RCT  Phase 3 | Global | 51.7 (12.0) | 0-12 | BARI 2mg | MTX: 72, GCs: N/A | 1.3 (3/229) | NR | Unclear |
|  |  |  |  |  | BARI 4mg |  | 1.3 (3/227) |  |  |
|  |  |  |  |  | PBO |  | 0.0 (0/228) |  |  |
|  |  |  |  | 12-24 | BARI 2mg |  | 0.4 (1/229) |  |  |
|  |  |  |  |  | BARI 4mg |  | 0.0 (0/227) |  |  |
|  |  |  |  |  | PBO |  | 0.0 (0/228) |  |  |
| Fleischmann 2017 (61)  (RA-BEGIN) | RCT  Phase 3 | Global | 50.3 (13.3) | 0-24 | BARI 4mg | GCs: 30 | 1.9 (3/159) | NR | Unclear |
|  |  |  |  |  | BARI 4mg + MTX | GCs: 39 | 1.4 (3/215) |  |  |
|  |  |  |  |  | MTX | GCs: 36 | 0.5 (1/210) |  |  |
|  |  |  |  | 24-52 | BARI 4mg |  | 0.6 (1/159) |  |  |
|  |  |  |  |  | BARI 4mg + MTX |  | 0.9 (2/215) |  |  |
|  |  |  |  |  | MTX |  | 0.5 (1/210) |  |  |
| Genovese 2016 (66)  (RA-BEACON) | RCT  Phase 3 | EU, LA, NA, SA, Asia | 55.7 (11.0) | 0-12 | BARI 2mg | MTX: 81 GCs: 53 | 1.2 (2/174) | NR | Unclear |
|  |  |  |  |  | BARI 4mg | MTX: 85 GCs: 54 | 2.3 (4/177) |  |  |
|  |  |  |  |  | PBO | MTX: 81 GCs: 66 | 0.6 (1/176) |  |  |
|  |  |  |  | 12-24 | BARI 2mg |  | 0.0 (0/174) |  |  |
|  |  |  |  |  | BARI 4mg |  | 0.0 (0/177) |  |  |
|  |  |  |  |  | PBO |  | 0.6 (1/176) |  |  |
| Keystone 2015 (62) | RCT  Phase 2b | NA, LA, EU | 51.0 (11.7) | 0-24 | BARI 2mg + MTX | GCs: 52 | 0.0 (0/52) | NR | High |
|  |  |  |  |  | BARI 4mg + MTX | GCs: 38 | 0.0 (0/52) |  |  |
|  |  |  |  |  | PBO + MTX | GCs: 52 | 0.0 (0/98) |  |  |
| Keystone 2018 (63) | Open-label LTEs |  | 53.0 (11.0) | 24-76 | BARI 4mg | MTX: 99, GCs: 51 | 5.6 (6/108) | IR 5.8 | Intermediate |
|  |  |  |  | 76-128 | BARI 4mg | MTX: 99, GCs: 46 | 1.3 (1/79) | IR 1.3 |  |
| Li 2020 (33)  (RA-BALANCE) | RCT  Phase 3 | Asia, LA | 49.2 (11.7) | 0-24 | BARI 4mg + MTX | GCs: NR | 2.1 (3/145) | NR | High |
|  |  |  |  |  | PBO + MTX |  | 0.7 (1/145) |  |  |
| Yang 2020 (35)  (substudy of RA-BALANCE) |  | China | 48.2 (11.7) | 0-24 | BARI 4mg |  | 1.7 (2/116) | NR | High |
|  |  |  |  |  | PBO |  | 0.9 (1/115) |  |  |
|  |  |  |  | 24-52 | BARI 4mg |  | 0.9 (1/116) |  |  |
|  |  |  |  |  | BARI 4mg switched from PBO |  | 0.0 (0/NR) |  |  |
| Tanaka 2016 (64) | RCT  Phase 2b | Japan | 56.8 (11.0) | 0-12 | BARI 2mg + MTX | GCs: 54 | 0.0 (0/24) | NR | Low |
|  |  |  |  |  | BARI 4mg + MTX | GCs: 75 | 0.0 (0/24) |  |  |
|  |  |  |  |  | PBO + MTX | GCs: 59 | 0.0 (0/49) |  |  |
| Tanaka 2018 (34) |  |  | 55.1 (11.9) | 12-62 | BARI 4mg + MTX | GCs: NR | 7.0 (5/71) | NR | High |
| Taylor 2017 (37)  (RA-BEAM) | RCT  Phase 3 | Global | 53.3  (5.3) | 0-24 | BARI 4mg + MTX | GCs: 56 | 1.4 (7/487) | NR | High |
|  |  |  |  |  | ADA^‖^ + MTX | GCs: 61 | 1.2 (4/330) |  |  |
|  |  |  |  |  | PBO^‖^ + MTX | GCs: 59 | 0.4 (2/488) |  |  |
|  |  |  |  | 24-52 | BARI 4mg + MTX |  | 0.8 (4/487) |  |  |
|  |  |  |  |  | ADA 40 mg Q2W + MTX |  | 0.3 (1/330) |  |  |
| **UPADACITINIB 15mg QD** | |  |  |  |  |  |  |  |  |
| Burmester 2018 (72)  (SELECT-NEXT) | RCT  Phase 3 | Global | 55.7 (11.9) | 12 | UPA | MTX: 76, GCs: 43 | 0.5 (1/221) | NR | Unclear |
|  |  |  |  |  | PBO | MTX: 66, GCs: 48 | 0.5 (1/221) |  |  |
| Fleischmann 2019 (32)  (SELECT-COMPARE) | RCT  Phase 3 | Global | 54.0 (12.0) | 0-26 | UPA + MTX | GCs: 60 | 0.8 (5/650) | NR | High |
|  |  |  |  |  | ADA + MTX | GCs: 62 | 0.3 (1/327) |  |  |
|  |  |  |  |  | PBO + MTX | GCs: 60 | 0.5 (2/652)^b^ |  |  |
| Fleischmann 2019 (71)  (SELECT-COMPARE) |  |  |  | 0-48 | UPA+ MTX |  | NR | ER 3.1 (2.2-4.2) | Unclear |
|  |  |  |  |  | ADA + MTX |  |  | ER 1.3 (0.5-2.8) |  |
| Fleischmann 2022 (29)  (SELECT-COMPARE) | open-label LTEs |  |  | 48-156 | UPA + MTX |  | 6.1 (87/1417) | ER 3.1 (2.5-3.8) | High |
|  |  |  |  |  | ADA + MTX |  | 2.1 (12/579) | ER 1.3 (0.7-2.2) |  |
| Pavelka 2020 (104)  (substudy of  SELECT-COMPARE) | RCT  Phase 3 | EU | 54.4 (11.9) | 0-26 | UPA + MTX |  | 0.0 (0/235) | NR | Unclear |
|  |  |  |  |  | ADA + MTX |  | 0.0 (0/122) |  |  |
|  |  |  |  |  | PBO + MTX |  | 0.0 (0/239) |  |  |
|  |  |  |  | 26-48 | UPA + MTX |  | NR | ER 2.3 (1.1-4.1) |  |
|  |  |  |  |  | ADA + MTX |  |  | ER 1.1 (0.1–4.0) |  |
|  |  |  |  |  | PBO + MTX |  |  |  |  |
| Genovese 2018 (73)  (SELECT-BEYOND) | RCT  Phase 3 | Global | 57.0 (11.4) | 0-12 | UPA | MTX: 85, GCs: 51 | 0.6 (1/164) | NR | Unclear |
|  |  |  |  |  | PBO | MTX: 83, GCs: 44 | 0.6 (1/169) |  |  |
|  |  |  |  | 12-24 | UPA |  | 1.3 (2/156) |  |  |
|  |  |  |  |  | UPA switched from PBO |  | 0.0 (0/72) |  |  |
| Kameda 2020 (74)  (SELECT-SUNRISE) | RCT  Phase 2b/3 | Japan | 55.2 (12.8) | 0-12 | UPA | MTX: 82, GCs: 57 | 0.0 (0/49) | NR | Low |
|  |  |  |  |  | PBO | MTX: 88, GCs: 49 | 2.0 (1/49) |  |  |
| Kameda 2021 (30)  (SELECT-SUNRISE) | blinded LTEs |  |  | 12-96 | UPA |  | 21.9 (14/64) | IR 12.3 | Unclear |
| [Rubbert-Roth 2020](https://pubmed.ncbi.nlm.nih.gov/?term=Rubbert-Roth+A&cauthor_id=33053283)(75)  (SELECT-CHOICE) | RCT  Phase 3 | Global | 55.6 (11.7) | 24 | UPA | MTX: 84, GCs: 56 | 1.3 (4/303) | NR | Unclear |
|  |  |  |  |  | ABA | MTX: 82, GCs: 51 | 1.3 (4/309) |  |  |
| Smolen 2019 (78)  (SELECT-MONOTHERAPY) | RCT  Phase 3 | Global | 54.9 (11.7) | 14 | UPA | MTX: 0, GCs: 53 | 1.4 (3/217) | NR | Unclear |
|  |  |  |  |  | MTX | GCs: 53 | 0.5 (1/216) |  |  |
| van Vollenhoven 2020 (77)  (SELECT-EARLY) | RCT  Phase 3 | Global | 52.6 (12.8) | 24 | UPA | MTX: 0, GCs: 46 | 2.2 (7/317) | NR | Unclear |
|  |  |  |  |  | MTX | GCs: 52 | 0.3 (1/314) |  |  |
| Takeuchi2021 (79)  (substudy of SELECT-EARLY) |  | Japan | 58.9 (11.3) |  | UPA | GCs: 41 | 7.4 (2/27) | NR | Unclear |
|  |  |  |  |  | MTX | GCs: 43 | 0.0 (0/28) |  |  |
| Zeng 2021 (76) | RCT  Phase 3 | China, Brazil,  South Korean | 51.7 (11.0) | 12 | UPA | MTX: 66, GCs: 67 | 1.8 (3/169) | NR | Unclear |
|  |  |  |  |  | PBO | MTX: 66, GCs: 66 | 0.6 (1/169) |  |  |
| **Psoriatic arthritis** | |  |  |  |  |  |  |  |  |
| **TOFACITINIB 5mg BID** |  |  |  |  |  |  |  |  |  |
| Gladman 2017 (80)  (OPAL Beyond) | RCT  Phase 3 | NA, LA, EU, Oceania  Asia | 49.3 (12.5) | 0-12 | TOFA | MTX: 75 GCs: 28 | 0.8 (1/131) | NR | Low |
|  |  |  |  |  | PBO | MTX: 77 GCs: 24 | 0.0 (0/131) |  |  |
|  |  |  |  | 12-24 | TOFA |  | 0.0 (0/131) |  |  |
|  |  |  |  |  | TOFA switched from PBO |  | 0.0 (0/66) |  |  |
| Mease 2017 (81)  (OPAL Broaden) | RCT  Phase 3 | NA,LA, EU, Asia, Oceania | 48.2 (12.1) | 0-12 | TOFA | MTX: 85 GCs: 27 | 0.9 (1/107) | NR | Low |
|  |  |  |  |  | ADA | MTX: 75 GCs: 22 | 0.0 (0/106) |  |  |
|  |  |  |  |  | PBO | MTX: 88 GCs: 17 | 0.0 (0/105) |  |  |
|  |  |  |  | 12-48 | TOFA |  | 0.9 (1/107) |  |  |
|  |  |  |  |  | ADA |  | 0.0 (0/106) |  |  |
|  |  |  |  |  | TOFA 5 or 10 mg BID switched from PBO |  | 0.0 (0/105) |  |  |
| Nash 2020 (82)  (OPAL Balance) | phase 3  Open-label LTEs | Global | 44.8 (11.8) | 0-144 | TOFA 5mg with adjustments to  10mg BID | MTX: 80 GCs: 19 | 2.8 (19/686) | IR 1.7 (1.0-2.6) | Intermediate |
|  |  |  |  |  | -average total daily dose 5mg BID |  | 2.5 (10/407) |  |  |
| Nash 2021 (124)  (OPAL Balance) |  |  |  | 0-192 | TOFA 5 or 10mg BID |  | 4.2 (29/686) | IR 1.7 (1.2-2.5) | Intermediate |
| Nash 2021 (83)  (substudy of OPAL Balance) | RCT  Phase 3 | EU, LA, NA, Asia, Oceania | 52.5 (11.2) | 48 | TOFA monotherapy | GCs: NR | 1.1 (1/90) | NR | Low |
|  |  |  |  |  | TOFA + MTX |  | 2.2 (2/90) |  |  |
| **UPADACITINIB 15mg QD** | |  |  |  |  |  |  |  |  |
| McInnes 2021 (85)  (SELECT-PsA 1) | RCT  Phase 3 | Global | 51.1 (12.1) | 24 | UPA | MTX: 70 GCs: 17 | 0.9 (4/429) | NR | Unclear |
|  |  |  |  |  | ADA | MTX: 67 GCs: 17 | 0.0 (0/429) |  |  |
|  |  |  |  |  | PBO | MTX: 70 GCs: 17 | 0.7 (3/423) |  |  |
| Mease 2020 (84)  (SELECT-PsA 2) | RCT  Phase 3 | Global | 53.6 (11.8) | 0-24 | UPA | MTX: 38 GCs: 10 | 1.4 (3/211) | NR | Unclear |
|  |  |  |  |  | PBO | MTX: 39 GCs: 11 | 0.9 (2/212) |  |  |
| Mease 2021 (86)  (SELECT-PsA 2) |  |  |  | 0-56 | UPA |  | NR | ER 3.8 (2.3-6.2) | Unclear |
| **Ankylosing spondylitis** | |  |  |  |  |  |  |  |  |
| **TOFACITINIB 5mg BID** |  |  |  |  |  |  |  |  |  |
| Deodhar 2021 (88) | RCT  Phase 3 | Global | 41.1 (11.5) | 0-16 | TOFA | MTX: 4 GCs: 10 | 0.0 (0/133) | NR | Unclear |
|  |  |  |  |  | PBO | MTX: 10 GCs: 5 | 0.0 (0/136) |  |  |
|  |  |  |  | 16-48 | TOFA |  | 2.3 (3/133) |  |  |
|  |  |  |  |  | TOFA switched from PBO |  | 1.5 (2/136) |  |  |
| van Der Heijde 2017 (87) | RCT  Phase 2 | NA, EU, Asia | 41.6 (11.6) | 16^††^ | TOFA | MTX: NR GCs: 4 | 0.0 (0/52) | NR | Unclear |
|  |  |  |  |  | PBO | MTX: NR GCs: 10 | 0.0 (0/51) |  |  |
| **UPADACITINIB 15mg BID** | |  |  |  |  |  |  |  |  |
| van Der Heijde 2019 (106)  (SELECT-AXIS 1) | RCT  Phase 2/3 | NA, EU, Asia, Oceania | 45.4 (12.5) | 0-14 | UPA | MTX: 4 GCs: 6 | 0.0 (0/93) | NR | Low |
|  |  |  |  |  | PBO | MTX: 2 GCs: 13 | 0.0 (0/94) |  |  |
| Deodhar 2022 (89) |  |  |  | 0-64 | UPA |  | 2.7 (5/182) | ER 2.1 | Unclear |
| **Ulcerative colitis** | |  |  |  |  |  |  |  |  |
| **TOFACITINIB 5mg or 10 mg BID** | |  |  |  |  |  |  |  |  |
| Sandborn 2012 (90)  (Induction) | RCT  phase 2 | EU, LA, Asia, SA | 42.9 (13.8) | 0-12 | TOFA 10mg | MTX: 0 GCs: 58 | 3.0 (1/33) | NR | Low |
|  |  |  |  |  | PBO | MTX: 0 GCs: 27 | 0.0 (0/48) |  |  |
| Sandborn 2017 (91)  (OCTAVE Induction 1) | RCT  Phase 3 | Global | 41.6 (14.7) | 0-8 | TOFA 10mg | MTX: 0 GCs: 45 | 0.6 (3/476) | NR | Unclear |
|  |  |  |  |  | PBO | MTX: 0 GCs: 48 | 0.8 (1/122) |  |  |
| Sandborn 2017 (91)  (OCTAVE Induction 2) | RCT  Phase 3 | Global | 40.8 (13.4) | 0-8 | TOFA 10mg | MTX: 0 GCs: 46 | 0.5 (2/429) | NR | Unclear |
|  |  |  |  |  | PBO | MTX: 0 GCs: 49 | 0.0 (0/112) |  |  |
| Sandborn2017 (91)  (OCTAVE Sustain) | RCT  Phase 3 | Global | 42.7 (14.1) | 8-60 | TOFA 5mg | MTX: 0 GCs: 51 | 1.5 (3/198) | NR | Unclear |
|  |  |  |  |  | TOFA 10mg | MTX: 0 GCs: 44 | 5.1 (10/196) |  |  |
|  |  |  |  |  | PBO | MTX: 0 GCs: 51 | 0.5 (1/198) |  |  |
| Sandborn 2022 (92)  (OCTAVE Open) | phase 3  Open-label LTEs | Global | 41.2 (14.0) | 60-240 | TOFA 5mg | MTX: 0 GCs: 1 | 7.4 (13/175) | IR 2.1 (1.1-3.6) | Intermediate |
|  |  |  |  |  | TOFA 10mg | MTX: 0 GCs: 28 | 7.8 (60/769) | IR 3.6 (2.7-4.6) |  |
| Sands 2019 (107)  (substudy of OCTAVE Open) |  | Global | 42.7 (13.8) | 60-108 | TOFA 5mg^¥^ | MTX: 0 GCs: 2 | 4.5 (3/66) | IR 2.3 (0.5-6.8) | Intermediate |
|  |  |  |  |  | TOFA 10mg* | MTX: 0  GCs: 7 | 12.3 (7/57) | IR 7.6 (3.0-15.6) |  |
| Vermeire 2021 (97)  (RIVETING Trial) | RCT  Phase 3b/4 | Global | 47.8 (13.8) | 0-24 | TOFA 5mg | MTX: 0  GCs: 0 | 1.4 (1/70) | IR 1.3 (0.0-6.9) | Low |
|  |  |  |  |  | TOFA 10mg |  | 4.3 (3/70) | IR 3.2 (0.7-9.4) |  |
| **UPADACITINIB 15mg, 30mg or 45 mg QD** | | |  |  |  |  |  |  |  |
| Sandborn 2020 (100) | RCT  Phase 2b | Global | 41.4 | 0-8 | UPA 15mg | MTX: NR GCs: 55 | 0.0 (0/49) | NR | Unclear |
|  |  |  |  |  | UPA 30mg | MTX: NR GCs: 48 | 0.0 (0/117) |  |  |
|  |  |  |  |  | UPA 45mg | MTX: NR GCs: 50 | 0.8 (1/123) |  |  |
|  |  |  |  |  | PBO | MTX: NR GCs: 54 | 0.0 (0/46) |  |  |

^a^mean (SD)

^b^In this group was also reported one varicella infection.

^C^ Incidence rate (IR) or Event rate (ER) (95% confidence interval) per 100 patient-years.

^§^ Reported at baseline.

^§§^ 1 HZ case. It was not reported whether this occurred in 5mg or 10mg BID group.

^†^6 weeks treatment & 6 weeks additional follow-up.

^††^8 weeks treatment & 4 weeks follow-up.

^‖^ Non-responders switched at week 16 to BARI 4mg QD.

^¥^De-escalation group: had previously received TOFA 10mg BID for induction therapy and were in remission after 52-week maintenance therapy with TOFA 10 mg BID.

*Escalation group: Had previously received TOFA 10mg BID for induction therapy and experienced treatment failure while receiving 5 mg BID maintenance therapy for 52 weeks.

SLR; systematic literature review, MTX; methotrexate, GCs; glucocorticoids, ADA; adalimumab, ABA; abatacept, ETN; etanercept, TOFA; tofacitinib, BARI; baricitinib, UPA; upadacitinib, PBO; placebo, IR; incidence rate, ER; event rate, RoB; risk of bias, RCT; randomized controlled trial, LTEs; ling-term extension study, NA; North America, US; United States, LA; Latin America, EU; Europe, SA; South America, SD; standard deviation, n; number of new events, N; number of patients at risk of developing HZ over a specific time period, %, proportion, BID; twice daily, QD; once daily, Q2W; every two weeks, N/A; not available, NR; not reported.

**Supplementary Table-3** Real-world studies included in this SLR. Study and patient characteristics, incident herpes zoster events and risk of bias.

| **Author, year (ref)** | **Study type** | **Region** | **Observational period, weeks^a^** | **Age, years^a^** | **Treatment groups** | **Concomitant**  **MTX and/or**  **GCs**^d^**, %** | **Herpes zoster** | | | **RoB** |
| --- | --- | --- | --- | --- | --- | --- | --- | --- | --- | --- |
|  |  |  |  |  |  |  | **Cumulative incidence, % (n/N)** | **Incidence rate^b^** | **Hazard ratio^c^** |  |
| **Rheumatoid arthritis** | |  |  |  |  |  |  |  |  |  |
| **Tofacitinib** |  |  |  |  |  |  |  |  |  |  |
| Bilgin 2021(46) | retrospective  cohort | Turkey | 46.4  (82.8) | 53.1 (12.6) | TOFA | MTX: 74, GCs: 25 | 5.4 (11/204) | 3.9 |  | High |
| Chen 2020(48) | retrospective  cohort | US | 36 | 57.8 (10.3) | TOFA | MTX: 51, GCs: 46 | 2.4 (15/634) | 3.7 (2.2-6.1) | 2.16 (1.09-4.28) | Low |
|  |  |  |  |  | ABA | MTX: 56, GCs: 44 | 1.3 (24/1785) | 1.7 (1.2-2.6) | 1.0 (reference) |  |
|  |  |  |  |  | TNFi | MTX: 71, GCs: 40 | 1.6 (93/5935) | 1.8 (1.4-2.1) | 1.48 (0.88-2.49) |  |
|  |  |  |  |  | RTX | MTX: 51, GCs: 53 | 2.6 (23/888) | 3.3 (2.2-5.0) | 1.82 (1.02-3.24) |  |
|  |  |  |  |  | TCZ | MTX: 53, GCs: 44 | 2.5 (19/759) | 3.4 (2.2-5.4) | 1.98 (1.06-3.68) |  |
| Chen 2020(57) | cohort | Taiwan | 81.6** | 56.8 (13.7) | TOFA | MTX: 65, GCs: 100 | 5.6 (7/125) | 3.6 (1.5-7.4) |  | Intermediate |
| Cohen 2018(60) | PMS | Global | NR | 60.0 | TOFA | NR | 7 events/9291  reports | NR |  | Intermediate |
| Curtis 2016(47) | cohort | US | NR | 60.1 (13.1) | TOFA | MTX: 39, GCs: 65 | 2.2 (38/1746) | 3.9 (2.8-5.3) | 2.01(1.40-2.88) | Low |
|  |  |  |  |  | TNFi | MTX: 56, GCs: 62 | 1.6 (618/38871) |  |  |  |
|  |  |  |  |  | GOL | NR | NR | 2.1 (1.5-2.9) | 1.09 (0.76-1.57) |  |
|  |  |  |  |  | IFX | NR | NR | 2.7 (2.3-3.1) | 1.17 (0.97-1.43) |  |
|  |  |  |  |  | ETN | NR | NR | 2.1 (1.8-2.5) | 1.06 (0.85-1.32) |  |
|  |  |  |  |  | ADA | NR | NR | 2.0 (1.7-2.3) | 1.0 (0.80-1.25) |  |
|  |  |  |  |  | CEZ | NR | NR | 2.6 (2.0-3.2) | 1.14 (0.87-1.48) |  |
|  |  |  |  |  | ABA | MTX: 47, GCs: 64 | 1.8 (209/11434) | 2.3 (2.0-2.7) | 1.0 (reference) |  |
|  |  |  |  |  | RTX | MTX: 44, GCs: 70 | 2.3 (110/4785) | 2.7 (2.2-3.2) | 1.12 (0.89-1.41) |  |
|  |  |  |  |  | TCZ | MTX: 44, GCs: 66 | 1.8 (115/6266) | 2.5 (2.1-3.0) | 1.12(1.40-2.88) |  |
| Curtis 2019 (41) | cohort | US | NR | 60.3 (12.6) | Total TOFA group |  | 2.8 (222/8030) | NR |  | Low |
|  |  |  |  |  | TOFA monotherapy |  | (73/NR) | 3.7 (2.9-4.6) | 1.0 (reference) |  |
|  |  |  |  |  | TOFA + MTX |  | (27/NR) | 3.4 (2.3-5.0) | 0.99 (0.64-1.54) |  |
|  |  |  |  |  | TOFA+ GCs |  | (82/NR) | 6.0 (4.9-7.5) | 1.75 (1.28-2.41) |  |
|  |  |  |  |  | TOFA + MTX + GCs |  | (40/NR) | 6.5 (4.8-8.8) | 1.96 (1.33-2.88) |  |
| Iwamoto 2017 (49) | cohort | Japan | 24 | 64.2 (11.5) | TOFA 5mg BID | MTX: 69, GCs: 53 | 7.1 (5/70) | NR |  | High |
| Kremer 2021(50) | prospective  cohort | US | 74.8 | 59.1 (12.6) | TOFA^*^ | MTX: 7, GCs: 68 | 2.1 (42/1999) | 1.4 (1.0-1.9) | 2.32 (1.43-3.75) | Low |
|  |  |  |  |  | bDMARDs | MTX: 11, GCs: 69 | 0.9 (78/8358) | 0.7 (0.5-0.8) | 1.0 (reference) |  |
| Mori 2019 (52) | prospective  cohort | Japan | 48.8 | 66.3 | TOFA 5mg BID for  1 year and then:  -withdrawal | MTX: 100, GCs: 11 | 5.3 (1/19) | NR |  | High |
|  |  |  | 69.2 |  | -continuation | MTX: 72, GCs: 28 | 16.7 (3/18) | NR |  |  |
| Pawar 2020 (51) | cohort | US | <48 | 54.5 (14.0) | TOFA | MTX: 66, GCs: 61 | (NR/1705) | 4.3 (3.6-5.0) | NR | Intermediate |
|  |  |  |  |  | ABA | MTX: 65, GCs: 61 | (NR/2831) | 2.7 (2.5-3.1) |  |  |
|  |  |  |  |  | ADA | MTX: 64, GCs: 61 | (NR/10205) | 2.2 (2.0-2.4) |  |  |
|  |  |  |  |  | CTZ | MTX: 64, GCs: 61 | (NR/2137) | 2.5 (2.1-3.0) |  |  |
|  |  |  |  |  | ETN | MTX: 64, GCs: 61 | (NR/8128) | 2.2 (2.1-2.4) |  |  |
|  |  |  |  |  | GOL | MTX: 64, GCs: 61 | (NR/1911) | 2.6 (2.1-3.1) |  |  |
|  |  |  |  |  | IFX | MTX: 65,GCs: 63 | (NR/2597) | 3.3 (2.9-3.6) |  |  |
|  |  |  |  |  | TCZ | MTX: 63, GCs: 62 | (NR/1079) | 2.4 (2.0-3.0) |  |  |
| **Baricitinib** |  |  |  |  |  |  |  |  |  |  |
| Peng 2020 (70) | PMS | Global | NR | 60.3% ≥50 | BARI | NR | 49 events/1598 reports | NR |  | High |
| Takahashi 2020 (69) | prospective  cohort | Japan | ≥24 | 66.1 (12.8) | BARI  2 or 4mg QD | MTX: 48, GCs: 40 | 6.2 (7/113) | 8.4 |  | Intermediate |
| Guidelli 2021 (67) | prospective  cohort | Italy | ≤48 | 59.0 (11.9) | BARI  4mg QD | MTX: 86, GCs: 73 | 1.3 (6/446) | NR |  | Intermediate |
| Tesei 2021 (68) | retro-prospective  cohort | Italy | 24 | 56.1 (11.2) | BARI  4mg QD | MTX: 37, GCs: 74 | 4.7 (2/43) | NR |  | Intermediate |
| **Tofacitinib or Baricitinib** | |  |  |  |  |  |  |  |  |  |
| Miyazaki 2021 (59) | cohort | Japan | 24 | 59.1 (13.4) | TOFA^¥^ | MTX: 73, GCs: 16 | 1.3 (2/156) | NR | NR | Intermediate |
|  |  |  |  |  | BARI^¥¥^ | MTX: 73, GCs: 17 | 3.6 (5/138) | NR |  |  |
| Iwamoto 2021 (58) | cohort | Japan | 24 | 66.5 (12.2) | TOFA 5mg BID | MTX: 68, GCs: 53 | 5.6 (9/161) | NR | NR | Intermediate |
|  |  |  |  |  | BARI 4mg QD | MTX: 46, GCs: 47 | 4.9 (4/81) |  |  |  |
| Choi 2022 (103) | retrospective  cohort | South Korea | 44 (16-116)  from JAKi initiation up to 1^st^ HZ event  48 (24-84)  follow-up after the 1^st^ HZ event | 60.2 (11.8) | TOFA or BARI | MTX: 67, GCs: 42 | 1^st^ episode  7.9 (33/416)  (22 events on TOFA and 11 on BARI users)  2^nd^ episode  1/29 | 4.8 |  | Intermediate |
| **Tofacitinib or Baricitinib or Upadacitinib** | | |  |  |  |  |  |  |  |  |
| Song 2022 (102) | case-control | Korea | NR | 55.2 (13.5) | JAKi (TOFA, BARI)^#^ | MTX: 81, GCs: 49 | 7.6 (17/223) | NR | NR | Low |
|  |  |  |  |  | bDMARDs^##^ |  | 4.8 (44/923) |  |  |  |
| Redeker 2022 (101) | prospective  cohort | Germany | ≤480 | 57.9 (12.5) | JAKi  (TOFA, BARI, UPA) | MTX: 37, GCs: 44 | 7.9 (56/713) | 2.2 (1.6-2.8) | 3.66 (2.38-5.63) | Intermediate |
|  |  |  |  |  | Anti-TNFs | MTX: 59, GCs: 60 | 3.3 (108/3242) | 0.9 (0.8-1.1) | 1.63 (1.17-2.28) |  |
|  |  |  |  |  | ETN | MTX: 51, GCs: 59 | 2.9 (73/2513) | 0.8 (0.7-1.1) | 1.28 (0.90-1.81) |  |
|  |  |  |  |  | ABA | MTX: 69, GCs: 57 | 4.2 (34/817) | 0.9 (0.6-1.2) | 1.45 (0.86-2.46) |  |
|  |  |  |  |  | RTX | MTX: 56, GCs: 68 | 4.3 (62/1431) | 1.0 (0.8-1.3) | 1.57 (1.03-2.40) |  |
|  |  |  |  |  | TCZ | MTX: 36, GCs: 58 | 5.0 (71/1424) | 0.9 (0.7-1.1) | 1.44 (0.99-2.11) |  |
|  |  |  |  |  | csDMARDs | MTX: 63, GCs: 47 | 3.5 (135/3851) | 0.7 (0.6-0.8) | 1.0 (reference) |  |
| **Ulcerative colitis** | |  |  |  |  |  |  |  |  |  |
| **Tofacitinib** |  |  |  |  |  |  |  |  |  |  |
| Avni-Biron 2022 (93) | retrospective  cohort | Israel | 29.6 (12-48) | 26.0 (14.8) | TOFA^‡^ | MTX: 0, GCs: 50 | 2.7 (2/73) | NR |  | High |
| Chaparro 2021 (99) | prospective  cohort | Spain | 44 (30-66) | 46.0 (1.3) | TOFA^‡‡^ | MTX: 4, GCs: 48 | 0.9 (1/113) | NR |  | High |
| Deepak 2021 (94) | retrospective  cohort | US | 24 (10.8-146) | 38.0 (11.0) | TOFA^¶^ | MTX: 0, GCs: 57 | 1.9 (5/260) | NR |  | Intermediate |
| Jameshorani 2021 (95) | prospective  cohort | Iran | 52 | 36.9 (12.5) | TOFA^¶¶^ | MTX: 0, GCs: 15 | 0.0 (0/53) | NR |  | Intermediate |
| Lair-Mehiri 2020 (98) | retro-prospective  cohort | France | 48 | 41.0 (12.0) | TOFA^§^ | MTX: 0, GCs: 0 | 7.9 (3/38) | NR |  | High |
| Straatmijer 2021 (96)(53) | retrospective  cohort | Netherlands | 60 | 45.0 (15.0) | TOFA^§§^ | MTX: 0, GCs: 42 | 5.6 (2/36) | NR |  | Intermediate |

^a^mean (SD)/median (IQR)

^b^Incidence rate (95% CI) per 100 patient-years.

^c^ Adjusted Hazard Ratio (95% CI).

^d^ Reported at baseline.

^*^88.6% received 5mg BID or 11mg QD.

** Patients were treated for ≥6 months.

^¥^ 89.7% received 10mg and 10.3% received 5mg BID.

^¥¥^ 88.4% received 4mg and 11.6% 2mg QD.

^#^ TOFA 71.7% and BARI 28.3%.

^##^ADA, ETN, IFX, GOL, ABA, TCZ and RTX.

^‡^8.2% received 5mg and 91.8% received 10mg BID.

^‡‡^ At baseline, 94% received 10mg BID and 5% 5mg BID. Later some patients were de-escalated from 10mg to 5mg BID and other were up-escalated from 5mg to 10mg BID.

^¶^ 5mg or 10mg BID or 11mg QD induction ± maintenance therapy. All patients were receiving 10mg BID at the time of VZV reactivation.

^¶¶^ 10mg BID for 8 weeks as induction therapy, followed by 5mg BID for 44 weeks as maintenance therapy.

^§^All patients started with 10mg BID for 12 weeks as induction therapy and then were treated with 10mg or 5mg BID for maintenance therapy. All events occurred in patients receiving 10mg BID.

^§§^Received 5mg or 10mg BID. One of the events occurred in a patient receiving 5mg BID and the other in a patients receiving 10mg BID.

SLR; systematic literature review, MTX; methotrexate, GCs; glucocorticoids, ADA; adalimumab, ABA; abatacept, TNFi; tumor necrosis factor inhibitor, RTX; rituximab, TCZ; tocilizumab, ETN; etanercept, IFX; infliximab, CEZ; certolizumab, CTZ; tocilizumab, bDMARDs; biologic disease modifying anti-rheumatic drugs, csDMARDs; conventional synthetic DMARDs, JAKi; Janus kinases inhibitors, TOFA; tofacitinib, BARI; baricitinib, UPA; upadacitinib, PBO; placebo, Abs; antibodies, HZ, CI; confidence interval, RoB; risk of bias, US; United States, PMS; post marketing surveillance, SD; standard deviation, n; number of new events, N; number of patients at risk of developing HZ over a specific time period, %, proportion, BID; twice-daily, QD; once daily, N/A; not available, NR; not reported

**Supplementary Table-4** Risk of bias in non-randomized studies according to the Newcastle-Ottawa scale.

| Study | Domains | | | Results | |
| --- | --- | --- | --- | --- | --- |
|  | **Selection** | **Comparability** | **Outcome** | **Score** | **Risk** |
| Avni-Biron 2022 | 2 | 0 | 1 | 3 | High |
| Bilgin 2021 | 2 | 0 | 1 | 3 | High |
| Chaparro 2021 | 2 | 0 | 1 | 3 | High |
| Chen 2020a | 3 | 2 | 2 | 7 | Low |
| Chen 2020b | 2 | 0 | 2 | 4 | Intermediate |
| Choi 2022 | 2 | 0 | 3 | 5 | Intermediate |
| Cohen 2018 | 2 | 0 | 2 | 4 | Intermediate |
| Curtis 2016 | 4 | 2 | 2 | 8 | Low |
| Curtis 2019 | 4 | 2 | 2 | 8 | Low |
| Deepak 2021 | 2 | 0 | 2 | 4 | Intermediate |
| Guidelli 2021 | 2 | 0 | 2 | 4 | Intermediate |
| Iwamoto 2017 | 2 | 0 | 1 | 3 | High |
| Ιwamoto 2021 | 3 | 1 | 2 | 6 | Intermediate |
| Jameshorani 2021 | 2 | 0 | 2 | 4 | Intermediate |
| Kremer 2021 | 3 | 2 | 2 | 7 | Low |
| Keystone 2018 | 2 | 0 | 3 | 5 | Intermediate |
| Lair-Mehiri 2020 | 2 | 0 | 1 | 3 | High |
| Miyazaki 2021 | 3 | 1 | 2 | 6 | Intermediate |
| Mori 2019 | 2 | 0 | 1 | 3 | High |
| Nash 2020 | 2 | 0 | 2 | 4 | Intermediate |
| Nash 2021 | 2 | 0 | 2 | 4 | Intermediate |
| Pawar 2020 | 3 | 2 | 1 | 6 | Intermediate |
| Peng 2020 | 1 | 0 | 1 | 2 | High |
| Redeker 2022 | 3 | 2 | 1 | 6 | Intermediate |
| Sands 2019 | 2 | 0 | 2 | 4 | Intermediate |
| Song 2022 | 3 | 2 | 2 | 7 | Low |
| Straatmijer 2021 | 2 | 0 | 2 | 4 | Intermediate |
| Takahashi 2020 | 2 | 0 | 2 | 4 | Intermediate |
| Tesei 2021 | 2 | 0 | 2 | 4 | Intermediate |
| Wollenhaupt 2019 | 2 | 0 | 2 | 4 | Intermediate |
| Yamanaka 2016 | 2 | 0 | 2 | 4 | Intermediate |
| Sandborn 2022 | 2 | 0 | 3 | 5 | Intermediate |

The Newcastle-Ottawa scale (NOS) judges on three broad aspects: the selection, the comparability of the study groups and the ascertainment either of the exposure or of the outcome of interest for case-control or cohort studies, respectively. Studies are appraised under an established 'star grading system' as having high (0-3 stars), intermediate (4-6 stars) or low (7-9 stars) RoB.


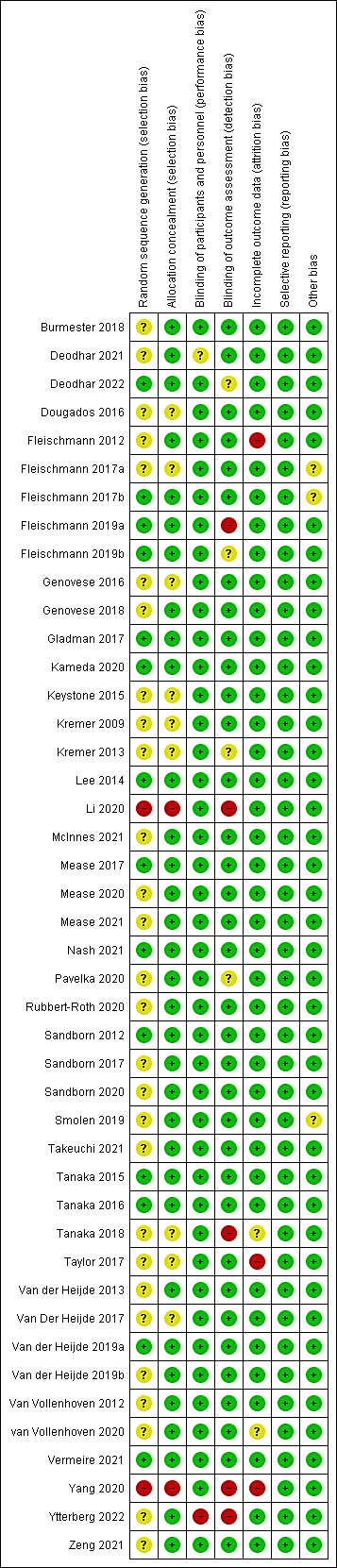

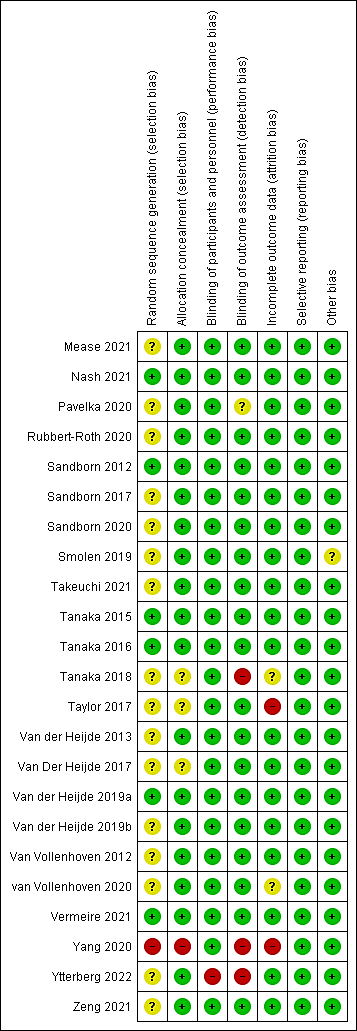


**Supplementary figure-1** Risk of bias in randomized studies according to the Cochrane RoB 2 tool.

Risk-of-Bias (ROB) 2 tool is structured into a set of 6 standard domains so as to identify selection, performance, detection, attrition and/or reporting bias, in addition to other specific biases that could be identified by the reviewers. Each domain is assigned with low, unclear or high RoB and the overall RoB judgment for each study is considered either as low (when all domains have low risk), as unclear (when up to three domains have unclear risk) or as high (when at least one domain has high RoB).
